# Perception, Pattern of Use, and Determinants of Family Planning Uptake Among Women of Childbearing Age in Ibadan, South West Nigeria

**DOI:** 10.21106/IJMA_47_2024

**Published:** 2026-07-06

**Authors:** Faith I. Olaniyi, Ayodele Ebenezer Owolabi, Gideon Temitope Olowe, Ololade Daniella Olunloyo, Eric Anuoluwapo Ogunleye, Ifeoluwapo O. Kolawole

**Affiliations:** 1 Faculty of Nursing Sciences, University of Ibadan, Ibadan, Oyo State, Nigeria; 2 Department of Public Health, University of Medical Sciences, Ondo State, Nigeria; 3 Department of Physiology, Faculty of Basic Medical Sciences, University of Medical Sciences, Ondo State, Nigeria; 4 Department of Nursing Sciences, Babcock University, Ogun State, Nigeria

**Keywords:** Barriers, Contraceptive Options, Factors, Reproductive Health, Utilization

## Abstract

**Background and Objective:**

While some efforts have been made to understand family planning practices as a vital component of reproductive healthcare, previous studies often lack specificity regarding the unique socio-cultural and geographical characteristics of the study area. The study, therefore, examined the perception, pattern of use, and determinants of family planning uptake among women of childbearing age (15–49 years) in a Local Government Area in Southwest Nigeria.

**Methods:**

A descriptive cross-sectional study was conducted using multi-stage sampling, with 190 respondents; data were analyzed using the Statistical Package for the Social Sciences (SPSS) version 25. Descriptive data were displayed in frequency tables. The mean and standard deviations were determined. The association between variables was tested using the chi-square at a 5% significance level (α = 0.05).

**Results:**

Among participants, 53% held positive perceptions of family planning. Less than half (47.9%) used family planning methods, and only 18.9% used barrier methods. Less than half (42.6%) had never visited family planning clinics, and less than half (49.5%) were unsure about how often they used family planning. Notably, more than one-third (35.3%) had used family planning methods for 1–5 years. Critical determinants of uptake included accessibility, adverse reactions, cost, and husband’s acceptance. There was no significant association between age (*p* = 0.090) or academic attainment (*p* = 0.569) and perception of family planning, nor between the current method of use (*p* = 0.09) and perception. However, injectable contraception was significantly associated with positive perception.

**Conclusion and Global Health Implications:**

The study highlights the need for targeted interventions to address barriers and enhance positive perceptions, particularly regarding access and affordability. Community-based education initiatives should prioritize increasing awareness and accessibility to a variety of contraceptive options, especially injectables, to meet the diverse reproductive health needs of women in the region.

## INTRODUCTION

### Background of the Study

Family planning allows individuals to achieve their desired family size and spacing of pregnancies through various methods, both natural and artificial.^[1]^ As a result, it is frequently used interchangeably with birth control. Known as child spacing, it is most commonly used by couples who want to regulate the number of children they have and the timing of their pregnancies.^[2]^ It encompasses various methods, such as sterilization and pregnancy termination.^[3]^ Natural and artificial methods for contraception include abstinence, coitus interruptus, safe periods, and lactational amenorrhea, as well as condoms, injectables, pills, and intrauterine contraceptive devices (IUCDs).^[4]^ The goal is to manage family child sizes, address population growth, and ensure equitable access to resources through responsible actions, education, and resource utilization.^[5]^ Family planning aims to achieve a desired family size, determine the timing of pregnancy, and promote economically viable intervals between childbirths.^[3]^ The primary goals include preventing health risks in pregnancies, reducing unsafe abortions, and lowering infant mortality rates. Family planning emphasizes the health and well-being of mothers and children by advocating for appropriate birth spacing.^[3]^ Even with the variety of techniques available, more than 200 million women in underdeveloped nations who want to avoid pregnancy do not use any form of contraception.^[4]^

Family planning has a long history, influenced by cultural, religious, and societal perspectives that often led to taboo practices due to perceived negative impacts on population growth, maternal and infant mortality rates, and societal well-being.^[6]^ The United Nations conferences in Bucharest (1974) and Mexico City (1975) significantly influenced 19th- and 20th-century global efforts to redefine family planning within the context of human rights, advocating for its wider acceptance.^[7]^ Despite these strides, Nigeria faces challenges in family planning, evident in its low contraceptive prevalence of 18% and a high fertility rate of 5.1.^[8]^ The 2018 Nigerian Demographic Health Survey revealed a 22% uptake of contraceptive use in Oyo State, despite donor support and organized family planning programs.^[9]^ This situation contributes to health challenges, with Nigeria ranking 187th out of 191 nations in health indicators.^[10]^ Low uptake of family planning methods in Nigeria may worsen health disparities, strain healthcare resources, and hinder progress towards improved maternal and child health. Factors like sociodemographics, economics, and healthcare influence contraceptive use. Despite extensive studies, a significant gap exists in understanding women’s perception, usage patterns, and determinants of family planning uptake.

This study investigates women’s perceptions, utilization, and uptake of family planning methods in Ibadan, Nigeria. It aims to understand the factors influencing decisions within a specific local government context and to inform targeted interventions and policy recommendations to enhance family planning services and outcomes in the area.

### Objectives of the Study

The objectives of this study were to assess the perceptions of women of childbearing age regarding family planning methods, determine their patterns of use, and identify the determinants influencing family planning uptake.

### Hypotheses

There is no significant association between respondents’ socio-demographic variables, such as age and educational qualifications, and their perception of family planning, nor between the methods respondents currently use and their perception of family planning.

## METHODS

### Study Setting and Study Design

This study was conducted in the Ibadan North Local Government Area (LGA) of Oyo State, Nigeria, covering three primary health care centers in Ibadan. Ibadan North LGA, located in Oyo State, Nigeria, is an urban center created in 1991 from the Ibadan municipal area.^[11]^ It covers 22 square kilometers and is bordered by five other LGAs: Akinyele, Lagelu, Egbeda, Ibadan Northwest, and Ibadan Northeast. The estimated population of Ibadan North LGA is approximately 330,399 inhabitants, with the majority being Yoruba.^[11]^ The LGA is home to notable landmarks, including the University of Ibadan and University College Hospital (UCH), making it a hub for education and healthcare services. Ibadan North LGA demonstrates significant variations in health indicators that influence family planning practices. The life expectancy in Nigeria is approximately 63.4 years, with urban areas like Ibadan North generally experiencing better health outcomes due to improved access to healthcare services.^[12]^ Despite this, maternal mortality remains high, with a national rate of 1,047 deaths per 100,000 live births in 2020, reflecting challenges in reproductive health.^[13]^ The demographic profile of Ibadan North shows a significant proportion of women of reproductive age (15–49 years), an important group for family planning programs. Because of the urban environment and easy access to educational facilities, women have a comparatively high literacy rate. Nonetheless, issues including disparities in income and societal perceptions of contraception continue to be major obstacles to the use of family planning. Reproductive health issues are made worse by common medical conditions, including starvation and malaria. These elements underscore the need for targeted initiatives to improve family planning knowledge and accessibility in the LGA. The researchers adopted a descriptive cross-sectional study design for data collection.

### Study Design

The researchers adopted a descriptive cross-sectional study design for data collection.

### Study Variables

The study assessed family planning uptake as the dependent variable (DV) and examined multiple independent variables (IVs) categorized into socio-demographic characteristics, perceptions of family planning, pattern of use, and determinants of uptake.

### Dependent Variable (DV)

Family planning uptake: Measured based on self-reported use of any family planning method (Yes/No).

### Independent Variables (IVs)

Socio-demographic characteristics: Assessed using 11 items, including age, marital status, education level, occupation, and number of children.

Perception of family planning: Measured using an 8-item questionnaire on a 4-point Likert scale (Strongly Agree to Strongly Disagree). Responses were scored, with a total mean ranging from 12 to 28. Participants scoring above the average mean score (24.88) were classified as having a positive perception, while those scoring below were classified as having a negative perception.

Pattern of use of family planning methods: Measured with six questions and analyzed using frequency distributions and percentages.

Determinants of family planning uptake: Assessed using 15 items that examined factors influencing use. Statistical analysis (univariate and Chi-square tests) was conducted to determine significant associations.

### Study Population

Women of childbearing age (15–49 years) attending selected centers in Ibadan, Nigeria, were enrolled in the study.

### Inclusion and Exclusion Criterion

The study included women of reproductive age (15–49 years) who provided consent to participate. However, women who met the inclusion criteria but were unable to take part due to unanticipated events, such as emergencies, last-minute scheduling changes on the day of data collection, or hospital visits that coincided with data collection, were excluded from the study.

### Sampling Techniques

The study used multi-stage sampling, divided into two stages.

**In stage one,** three primary health centers were selected using simple random sampling. The selected primary health centers, Agbowo, Sango, and Idi-Ogungun, were chosen from 21 primary health centers in the Ibadan North LGA. The average number of women at each primary health center was obtained from the register to determine the sample size. This step ensured that the sample size accurately reflected the population of women seeking healthcare services at each primary health center. **In stage two,** individual participants were randomly selected from the registers of the selected primary health centers and recruited into the study, ensuring fair representation of the diverse population within the chosen centers.

### Sample Size Calculation

The standard sample size formula (Finite Population Correction Factor) was used.^[14]^


$$\left[{\text{z}}^{2}*\text{p}\left(1-\text{p}\right)\right]/{\text{e}}^{2}/1+\left[{\text{z}}^{2}*\text{p}\left(1-\text{p}\right)\right]/{\text{e}}^{2}*\text{N}$$

- N is the population size
- z is the z-score
- e is the margin of error
- p is the standard deviation

Formula 1: Sample size for an infinite population


$$\text{S}={\text{z}}^{2}*\text{p}*\left(1-\text{p}\right)/{\text{M}}^{2}$$

Formula 2: Adjusted sample size

Adjusted sample size = (S)/1+(S–1)/Population

Where;

- S = sample size for infinite population
- Z = Z score = 2.58
- P = population proportion (0.5)
- M = Margin of error (0.05)
- Population = 266
- Confidence level = 99%

Z = 2.58, P = 0.5, M = 0.05

S = Z^2^ ⁎ P ⁎ (1–P) / M^2^

S = (2.58)2 ⁎ (0.5) ⁎ (1–0.5) / (0.05)^2^

S = 665.64

Adjusted sample size = (S)/1+(S–1)/Population

Sample size = (665.64)/1+(665.64–1)/266

**Sample size:** 190.256425

**Therefore, the sample size is approximately 190.**

### Study Instrument

A modified questionnaire, titled the Family Planning Perception and Utilization Questionnaire (FPPUQ), was used. It was developed by combining questions from various articles^[15–17]^ into a unified tool. The authors adopted the instrument after conducting a thorough literature review to ensure its applicability to the study’s objectives and context. It comprised four distinct sections: Section A comprises eleven (11) items assessing respondents’ socio-demographic data. Section B contains eight (8) questions designed to evaluate perceptions of family planning methods. Section C includes six (6) items examining the pattern of use of family planning methods. Finally, Section D features fifteen (15) items assessing the determinants of family planning uptake.

#### Validity and reliability of the instrument

The study involved experts in reproductive, maternal, and child health to modify an instrument to meet study objectives. The instrument was translated from English into Yoruba to facilitate participant comprehension. The instrument was updated in response to reviewers’ comments and the pretest results. The preliminary study clarified questions and the sequence before final administration. Data analysis confirmed the instrument’s internal consistency, with a Cronbach’s alpha value of 0.7. This suggests that the terms included in the instrument are correlated with each other, indicating instrument reliability in measuring the intended constructs. 

### Procedure for Data Collection

The Oyo State Ethics Review Committee at the Ibadan North Local Government Secretariat granted ethical approval for the study, which involved distributing 190 questionnaires to participants. Prior to data collection, the primary healthcare administrators were contacted in order to evaluate their centers’ registers and calculate the number of participants. At this point, the study’s goal was provided. Following that, the administrators received a more thorough presentation of the study’s goals, procedures, and ethical approval. Following further explanation, the administrators approved moving forward with the study. Informed consent was obtained from each participant prior to the distribution of the surveys. They were given an informed consent form to read, and those who agreed to participate signed it. Details regarding the study were given to the respondents, and those who did not consent were excluded. Data collection lasted for 3 weeks, ensuring the majority of respondents had the opportunity to participate. The questionnaires were checked for completeness and proper filling, ensuring a comprehensive and accurate study.

### Method of Data Analysis

Data were cleaned and coded using the Statistical Package for the Social Sciences (SPSS) version 25 and analyzed using descriptive statistics, including frequency counts, percentages, mean, and standard deviation, to answer the research questions.^[18]^ Respondent’s socio-demographic characteristics were presented using percentages and frequency distribution tables. Respondents’ responses on perception were provided using a 4-point Likert scale of strongly agree, agree, disagree, and strongly disagree. The scale was scored as 3 = strongly agree, 2 = agree, 1 = disagree, and 0 = strongly disagree for positive statements, and reverse-coded for negative statements. Respondents’ total mean range is between 12 and 28, and participants with a mean score above the average of 24.88 were adjudged to have a positive perception toward family planning, whereas those with a mean score below 24.88 were adjudged to have a negative perception. Respondent’s patterns of use of family planning methods were categorized and presented using frequency tables and percentages. Their pattern of family planning method use was determined from their responses to six questions. The determinants of the use of family planning methods were assessed using univariate analysis to evaluate the impact of the variables on the use of family planning methods. The chi-square test was used to test the hypotheses at a 5% significance level (α = 0.05). This established a significant association between the variables of interest, if any.

### Ethical Considerations

The study was approved by the University of Ibadan/UCH Ethics Review Committee (approval number: UI/EC/24/0046) and the Oyo State Ethics Review Committee, and permission was obtained from the heads of selected primary healthcare centers. Participants voluntarily agreed to participate and provided informed consent. Participants were assured that all data provided would be kept confidential. Anonymity was strictly maintained, and participants could trust that the data collected would be used solely for academic purposes. Personal details such as names, addresses, and phone numbers were not collected to maximize the anonymity of the data obtained. The informed consent form was translated from English into Yoruba by a member of the research team for inclusivity. The study did not involve invasive procedures, no harm was caused, and participants could withdraw at any time without consequences.

#### Statement of compliance

This manuscript complies with the Strengthening the Reporting of Observational Studies in Epidemiology (STROBE) guidelines for observational studies.

## RESULTS

### Sociodemographic Characteristics of Study Participants

Most study participants were between 20 and 29 years old (46.3%), with a mean age of 29 ± 8 years. Many of them (62.1, 50.5%) had secondary education. Some (33.2%) of the respondents were married for 1–5 years. Meanwhile, 60% of the respondents had between 1 and 4 children alive, with 37.5% having a 2-year interval between the children.

### Perception of Family Planning Methods Among Women

As shown in Table 1, 43.2% agreed that contraceptive methods are advantageous, while 51.6% believed family planning is essential. Moreover, 37.9% felt comfortable with contraceptives, and 47.4% considered them effective. Furthermore, 49.5% agreed that contraceptive methods have advantages for the mother, and 40% agreed that, culturally, contraceptives are good. However, 51.1% agreed that contraceptives are a Western idea and not necessary. Notably, 51.1% strongly agreed that both partners should decide the use of contraceptives. More than half (53%) had positive perceptions, and 47% had negative perceptions.

**Table 1:** Perception of family planning methods among participants.

Variables	Strongly disagree	Disagree	Agree	Strongly agree
	Frequency (%)	Frequency (%)	Frequency (%)	Frequency (%)
Contraceptives have advantages for the family	6 (3.2)	25 (13.2)	82 (43.2)	77 (40.5)
Family planning is important	7 (3.7)	15 (7.9)	70 (36.8)	98 (51.6)
Feel comfortable with contraceptives	9 (4.7)	41 (21.6)	68 (35.8)	72 (37.9)
Contraceptives are effective	9 (4.7)	23 (12.1)	90 (47.4)	68 (35.8)
Contraceptives have advantages for the mother	6 (3.2)	28 (14.7)	94 (49.5)	62 (32.6)
Culturally, contraceptives are good	8 (4.2)	59 (31.1)	76 (40)	47 (24.7)
Contraceptives are a Western idea adopted by us and are not necessary*	26 (13.7)	32 (16.8)	97 (51.1)	35 (18.4)
Both partners should decide on the use of contraceptives	6 (3.2)	11 (5.8)	76 (40)	97 (51.1)

Key: Likert scale: 0 = Strongly disagreed, 1 = Disagreed, 2 = Agreed, and 3 = Strongly agreed.*Reverse coded.

### Patterns of Use of Family Planning Methods Among Women

The study found that two-thirds (66.8%) of respondents have used a family planning method before. Less than half (47.9%) are currently using family planning methods, including barrier methods (18.9%). About half (42.6%) have never visited the family planning clinic, and 49.5% cannot say the frequency of the family planning method they used. About one-third (35.3%) of them have a 1–5 year history of family planning use [Table 2]. These data indicate that current usage rates, especially for barrier methods, are lower, highlighting potential gaps in ongoing access and use of family planning services.

**Table 2:** Patterns of use of family planning methods.

Variables	Categories	Frequency (*n* = 190)	Percent (%)
Ever used any family planning methods?	No	63	33.2
	Yes	127	66.8
Currently using any family planning methods?	No	99	52.1
	Yes	91	47.9
If yes, please tick the method(s):	Abstinence	3	1.6
	Barrier method	36	18.9
	Contraceptive pills	8	4.2
	Emergency contraceptive	4	2.1
	Hormonal implant method	9	4.7
	Injectables	13	6.8
	Intra-uterine contraceptive device	13	6.8
	Withdrawal method	5	2.6
Last visit to a family planning clinic?	Never visited	81	42.6
	Within the last 1 month	11	5.8
	Within the last 3 months	18	9.5
	Within the last 6 months	49	25.8
	Others	31	16.3
Frequency of use of any family planning method	Cannot say	94	49.5
	When affordable	25	13.2
	Consistent	71	37.4
Duration of use of any family planning method	<6 months	50	26.3
	6 months to <12 months	47	24.7
	1–5 years	67	35.3
	>5 years	26	13.7

### Determinants of Family Planning Methods

More than half agreed that availability of facilities/equipment (51.6%), lack of awareness (52.6%), effectiveness of family planning (51.6%), religious acceptance (53.7%), husband’s acceptance (58.9%), cost of contraceptives (63.2%), and adverse reactions (64.7%) are determinants of uptake of family planning methods. Moreover, more than two-thirds (70.5%) agreed that accessibility of services is a determinant of uptake of family planning methods. Based on the mean score, the main significant determinants of uptake of family planning methods are accessibility of family planning services (1.71 ± 0.46), adverse reactions (1.65 ± 0.48), cost of contraceptives (1.63 ± 0.48), and husband’s acceptance (1.59 ± 0.49) [Table 3].

**Table 3:** Determinants of family planning methods.

Variables	No f (%)	Yes f (%)
Attitude of family planning provider	96 (50.5)	94 (49.5)
Availability of facilities/equipment	92 (48.4)	98 (51.6)
Lack of awareness	90 (47.4)	100 (52.6)
Language barrier	127 (66.8)	63 (33.2)
Schedule of the family planning clinic	103 (54.2)	87 (45.8)
Effectiveness of family planning method	92 (48.4)	98 (51.6)
Accessibility of family planning services	56 (29.5)	134 (70.5)
Cultural acceptance	100 (52.6)	90 (47.4)
Religious acceptance	88 (46.3)	102 (53.7)
Husband’s acceptance	78 (41.1)	112 (58.9)
Lack of informed choice	128 (67.4)	62 (32.6)
Cost of contraceptives	70 (36.8)	120 (63.2)
Adverse reactions	67 (35.3)	123 (64.7)
Fear of sin	116 (61.1)	74 (38.9)

0 = No; 1 = Yes.

### Socio-demographic Perception of Family Planning

There is no significant relationship between age (*x*^2^ = 6.492, *p* = 0.090) and academic attainment (*x*^2^ = 1.155, *p = *0.569), with respect to their perception of family planning at *p *< 0.05. However, being <20 years old and having a primary school education are significantly associated with a positive perception of family planning.

### Current Family Planning Methods Used and Their Perception

There is no significant relationship between the method of family planning currently in use (*x*^2^ = 6.492, *p* = 0.09) and their perception of family planning at *p *< 0.05. However, using the injectables method of family planning is significantly associated with a positive perception of family planning.

## DISCUSSION

The average age of respondents in this study was 29 ± 8 years, consistent with a study [19] reporting a mean age of 29 ± 6 years. They also had secondary education; this finding is consistent with a study [15], which found that about half of the participants had secondary education.^[20]^

The study found that participants held positive perceptions of family planning, consistent with findings from,^[21]^ where a higher proportion of women perceived contraceptive use as essential and were satisfied with the contraceptive services they received at medical centers. These findings highlight the generally positive attitudes toward family planning in our study area, contributing to a broader understanding of contraceptive acceptance and utilization patterns. However, cultural and social nuances might also influence these perceptions, as discussed further below.

Interestingly, while some participants reported current use of family planning methods, a smaller proportion preferred barrier methods. This contrasts with a study^[22]^ examining contraceptive preferences among Nigerian women, which found that the majority favored the Jadelle implant, with Norplant the least preferred. These differences may reflect varying socio-economic backgrounds, cultural beliefs, or the accessibility of family planning resources across different regions. For instance, barrier methods might be favored in this population due to lower costs or cultural concerns about long-term contraceptive methods. This variation underscores the importance of region-specific research to tailor family planning interventions effectively.

The study identified accessibility to family planning services, adverse reactions, contraceptive costs, and husband’s acceptance as significant determinants of contraceptive uptake. While these findings are similar to previous research,^[23]^ which highlighted informed choice, cost, and access as critical factors, the emphasis on husbands’ acceptance in our study points to cultural influences that may shape family planning decisions. In this population, male partners often play a significant role in reproductive health decisions, which may explain why husbands’ acceptance is a prominent factor. This highlights the need for culturally sensitive approaches to promoting family planning services, especially in areas where partner approval is crucial for contraceptive uptake. Findings further revealed that there is no significant relationship between age and academic attainment, respectively, and their perception of family planning. However, being <20 years and having a primary school education are significantly associated with a positive perception of family planning. This finding contrasts with a report, ^[24]^ in which women aged 30 years and older, as well as those with secondary education or higher, had a more positive perception of family planning. 

Lastly, the study found no significant relationship between the method of family planning currently in use and the general perception of family planning. However, the use of injectables was associated with more positive attitudes, suggesting that method choice may influence perceptions to some extent. Targeted education and counseling efforts could highlight the benefits of injectables and address concerns about them to further enhance positive perceptions and uptake of family planning methods.

### Cultural and Social Influences on Family Planning

This population’s perceptions and practices are likely influenced by cultural conventions surrounding family planning. Our research on spouse acceptability as a determinant is consistent with the social norms that frequently drive men’s participation in family planning decisions. A preference for barrier methods over hormonal or implant options may also result from religious beliefs in the studied area that influence opinions about particular contraceptive methods. In addition to making family planning programs more respectful and relevant to the needs and beliefs of the local community, considering these cultural aspects may also increase their efficacy.

### Limitations of the Study

Although the design provided valuable insights into perceptions and patterns of family planning uptake, it was limited in assessing the dynamic influences of factors such as male involvement over time. This limitation underscores the importance of a future study exploring the impact of male involvement on family planning decisions. A comprehensive sampling strategy was implemented to ensure a diverse representation of women of childbearing age within Ibadan North Local Government. Additionally, the questionnaire was carefully designed and validated to accurately capture perceptions, patterns of use, and determinants of family planning uptake. Although the small sample size was a limitation, rigorous sampling methods and data analysis techniques were employed to maximize the generalizability of the findings within the study context. Future research could adopt a longitudinal design to track changes over time and address this limitation by monitoring contraceptive outcomes, thereby enabling a better understanding of satisfaction and continuation rates. This would help identify opportunities to enhance patient-centered care and tailor family planning services to meet women’s long-term needs.

## CONCLUSION AND GLOBAL HEALTH IMPLICATIONS

The study highlights the vital role that midwives and community health workers play in advancing reproductive health globally by incorporating up-to-date information into teaching initiatives. It aims to prepare future midwives and health workers with the skills needed to guide women in making informed decisions about family planning, ensuring access to and uptake of services. The study also identifies research gaps and suggests areas for further exploration for effective global interventions. To maximize their impact, midwives and community health workers should advocate for equitable access to family planning resources and work with policymakers to develop culturally sensitive, accessible services. In many settings, these healthcare professionals are integral to local healthcare delivery, particularly through community-based programs and initiatives to engage male partners. Such targeted interventions can help overcome barriers to family planning and enhance reproductive health outcomes globally, ensuring that women have the necessary resources for informed and empowered choices.

### Key Messages

- Major barriers to family planning include accessibility, cost, and husband’s acceptance, highlighting the need for targeted community education to improve awareness and access, especially for injectable contraception.
- The importance of involving both partners in contraceptive decision-making cannot be overemphasized.
- The discrepancies observed between this study and previous findings suggest the importance of context-specific research to tailor interventions effectively and address challenges.

**Acknowledgments: **Special thanks to all the staff and management of the primary health centers for their support during the period of data collection.

## COMPLIANCE WITH ETHICAL STANDARDS

### Conflicts of Interest

The authors declare no competing interests.

### Financial Disclosure

Nothing to declare.

### Ethics Approval

Ethical approval was obtained from the University of Ibadan/University College Hospital Ethics Committee with registration number: NHREC/05/01/2008a and assigned number UI/EC/24/0046. The study also obtained approval from the Oyo State Ethics Review Committee. Informed consent was obtained from the participants.

### Disclaimer

None.

### Declaration of Patient Consent

The authors confirm that all appropriate patient consent was obtained prior to the inclusion of any patient-related information in this manuscript.

### Use of Artificial Intelligence (AI)-Assisted Technology for Manuscript Preparation

The authors confirm that there was no use of Artificial Intelligence (AI) Assisted Technology for assisting in the writing or editing of the manuscript, and no images were manipulated using AI.
